# Rapamycin in preventive (very low) doses

**DOI:** 10.18632/aging.100645

**Published:** 2014-03-22

**Authors:** Roman V. Kondratov, Anna A. Kondratova

**Affiliations:** ^1^Center for Gene Regulation in Health and Diseases, BGES, Cleveland State University, Cleveland, OH 44115; USA; ^2^ Department of Molecular Genetics, Lerner Research Institute, Cleveland Clinic, Cleveland, OH 44195; USA

In the recent paper of Popovich et al. published in Cancer Biology and Therapy [1], positive effects of low doses of rapamycin on survival of caner-prone HER-2/neu mice have been reported. Inhibitors of the mTOR signaling pathway are already widely employed for anticancer therapy in humans. At the same time, over several last years a substantial body of evidence suggesting an anti-aging effect of at least one mTOR inhibitor, rapamycin, was accumulated. For example, rapamycin delayed death without changing the distribution of presumptive causes of death in genetically heterogeneous mice [2] and extended lifespan of cancer-prone mice, such as transgenic HER-2/neu mice [3]. Thus, a question was raised whether lifespan extension by rapamycin is a consequence of the anti-cancer effect of rapamycin (such as suppression of tumor initiation or slowing down tumor progression) or a direct result of its anti-aging properties (or both). Several studies supported effectiveness of rapamycin in delaying of aging independently from its anti-cancer activities. For example, Wilkinson et al. [4] demonstrated that rapamycin treatment significantly decreased numerous manifestations of aging and alleviated age-dependent decline in spontaneous activity in 20-22 month genetically heterogeneous mice (the age when the majority of mice survived). In the study of Anisimov et. al. [5] conducted with inbred female mice, rapamycin treatment extended lifespan, inhibited age-related weight gain, and increased the percentage of mice having regular estrous cycle at 18 months. However, the question whether lifespan extension was in part a result of suppression of cancer was not answered in these studies. A recent work from our laboratory, conducted on *Bmal1-/-* mice suffering from premature aging, provides an example of lifespan extension induced by rapamycin which is not due to inhibition of neoplastic diseases [6]. Strikingly, these mice, in spite of having many prominent aging phenotypes, virtually never develop cancer, and die from systemic failure due to progressive degeneration of nervous and cardiovascular and muscle systems. We found that rapamycin treatment extended lifespan of *Bmal1-/-* mice from 8 to 12 months. Activity of mTORC1 in tissues of *Bmal1-/-* was highly elevated, thus, treatment with rapamycin extended their lifespan mostly likely through suppression of mTOR signaling.

Therefore, this study helps to further separate the life-extending effect of rapamycin from its cancer-preventing properties. Taken together, these findings indicate that rapamycin can be considered as a good candidate for a preventive anti-aging medicine.

Using a substance as a preventive medicine immediately raises questions about its overall safety and potential side effects; these questions are even more principle here compared with the situation of disease treatment. For example, in cancer treatment the drugs are used under conditions when the disease has already been developed and is deadly dangerous. Therefore, administration of high doses of drugs (even in spite of particular side effects) is justified; additionally, drugs are often administered during relatively short periods of time. On the contrary, prevention suggests chronic exposure to a medicine before the actual disease is developed, which may not happen whatsoever even without taking the preventive medication (considered as a disease, aging is an apparent exception). Thus, an ideal chemopreventive medicine should not have any side effects. Rapamycin, like any other existing medicine, does have side effects. In addition to well-known side effects of high doses of rapamycin in humans [7], life-long chronic exposure to rapamycin, while preventing most age-related diseases and extending healthspan, was shown to increase incidence of cataracts and some other alterations in mice [4]. This type of known and potential side effects makes consideration of rapamycin as a chemopreventor much less attractive for a healthy individual. Several groups noticed another side effect, a general decrease in animal robustness, when treatment with rapamycin was started in very young mice [1, 8]; this underscores the importance of mTOR pathway activity during development and points out to the significance of another variable, the onset of treatment, in diminishing of side effects. Therefore, the question about safe dosage, timing and mechanisms of delivery of rapamycin is extremely important.

Popovich and colleagues reported the lifespan extension activity of low doses of rapamycin administered in the intermittent manner (with two-week breaks between two-week-long bi-daily treatments) in a mouse model of breast cancer. In this model, mammary carcinoma develops in female mice overexpressing oncogene HER2; cancer advances very fast with average lifespan of about 9 months. Different regimes of treatment with low doses of rapamycin allowed increasing lifespan with or without significant cancer prevention (correlating with the age the treatment started). The present study is an extension of the previous work by this team of collaborators on anticancer/longevity effects of rapamycin given in doses corresponding to the therapeutic oral dose in humans in the same mouse model [3]. There, rapamycin reduced cancer incidents, suppressed development of tumors, and extended lifespan by almost two fold. The positive effect of low doses of rapamycin on longevity in the follow-up study supports previous observations. The most important advantage of the new study derives from the significant reduction of the dose used. The above-discussed side effects are generally a matter of dose: indeed, at particular doses any substance, even water, turns out to be toxic; thus, the ability of rapamycin to work at low doses makes it substantially more attractive as a candidate for a preventive medicine.

In conclusion, we would like to accentuate that indeed anti-cancer and anti-aging activities of rapamycin can be separated. In the case of HER2-overexpressing mice, whether rapamycin works through the delay of aging or other mechanisms are involved still has to be found. At the same time, it also supports the strategy to use rapamycin in combination with other anticancer drugs. Indeed, even if rapamycin on its own would not affect tumor, it still might improve survival and therapeutic outcome. It is worth testing at least in other animal models.
